# Lethal dog attacks on adult rhesus macaques (*Macaca mulatta*) in an anthropogenic landscape

**DOI:** 10.1007/s10329-024-01122-y

**Published:** 2024-03-06

**Authors:** Bidisha Chakraborty, Krishna Pithva, Subham Mohanty, Brenda McCowan

**Affiliations:** 1 Department of Population Health and Reproduction, School of Veterinary Medicine, University of California, Davis, CA USA; 2 California National Primate Research Center, University of California, Davis, CA USA

**Keywords:** Dog–nonhuman primate interactions, Dog attack, Anthropogenic, Rhesus macaques, Urban ecology, Dog monkey interactions

## Abstract

For nonhuman primates living in anthropogenic areas, predation by larger predators is relatively rare. However, smaller predators, such as free-ranging as well as domesticated dogs, can shape the socioecology of urban nonhuman primates, either directly by attacking and killing them or indirectly by modifying their activity patterns. Here, we describe three (two probably fatal) cases of dog attacks on adult rhesus macaques inhabiting an anthropogenic landscape in Northern India and the circumstances surrounding these incidents. We discuss the importance of considering human presence and intervention in dog–nonhuman primate relationships while studying nonhuman primate populations across anthropogenic gradients, and its potential influences on group social dynamics and transmission of zoonotic agents.

**Supplementary Information:**

The online version contains supplementary material available at 10.1007/s10329-024-01122-y.

## Introduction

Owing to human-induced habitat changes, nonhuman primate species face the risk of local extinction or are adapting to living in proximity to humans by becoming more ecologically flexible (Hockings et al. 2012; Hansen et al. 2022). Living near humans entails various costs, including increased aggression from both conspecifics and humans (McCarthy et al. 2009; Hetman et al. 2019), accidental deaths (Pereira et al. 2020), and heightened stress levels (Maréchal et al., 2011). Though the largely predictable nature of high-quality anthropogenic food can positively affect individual fitness (Kurita et al. 2008), anthropogenic food such as garbage and junk food can also negatively affect animal cardiovascular health (Hannah et al. 1991).

A potential advantage for animals living in human-impacted areas is believed to be reduced risk from large predators, such as tigers (*Panthera tigris*) and leopards (*Panthera pardus*) (Crooks and Soulé 1999). However, lack of such top-down competition positively affects the population of mesopredators, such as coyotes (*Canis latrans*) and dogs (*Canis lupus familiaris*), which might affect their prey species (Takimoto and Nishijima 2022). As one of most abundant carnivores across the globe, dogs pose significant threats to wildlife conservation by disturbing, competing with, and predating upon vulnerable wildlife (Gompper 2014). Human behaviors, such as domestication and training of dogs for livestock and crop protection as well as hunting, can directly affect such dynamics in anthropogenic areas (Waters et al. 2023). Interestingly, dogs not only directly affect local wildlife by predation, but can also affect their feeding and movement behavior simply by their presence or by competing for food and space (Waters et al. 2023). Given their coexistence with both humans and wildlife, dogs can transmit diseases, for example, rabies, potentially leading to cross-species infections (Gautret et al. 2014; Kumar Bharti 2016). However, despite dogs’ increasingly significant effects on nonhuman primate behavior and survival, there are few descriptions of dog–nonhuman primate interactions in anthropogenic areas.

Rhesus macaques (*Macaca mulatta*) have been able to thrive in human-impacted landscapes (Cooper et al. 2022). Large predators of rhesus macaques, such as tigers and leopards, usually avoid dense urban areas (Carter et al. 2015). However, aggression between rhesus macaques and smaller predators such as dogs is commonly observed, and predation on rhesus macaque infants and unknown individuals by dogs has been reported (Anderson 1986; Chetry et al. 2005). There are several cases of dogs preying on or harassing other nonhuman primates species, including brown howler monkeys (*Alouatta guariba*) (da Silva et al. 2021), black howler monkeys (*Aloutta pigra*) (Franquesa-Soler et al. 2023), Japanese macaques (*Macaca fuscata*) (Hill 2014), young female and infant barbary macaques (*Macaca sylvanus*) (Waters et al. 2017), juvenile long tailed macaques (*Macaca fascicularis*) (Riley et al. 2015), and Central Himalayan langurs (*Semnopithecus schistaceus*) (Nautiyal et al. 2023), among others (see an early review by Anderson 1986, and recent review by Waters et al. 2023). Perhaps surprisingly, there are few published reports about rhesus macaque–dog interactions (Anderson 1986; Chetry et al. 2005), yet detailed records of such interactions are needed to understand how dogs might shape the socioecology of nonhuman primates in human-impacted landscapes (Gompper 2014). In this report, we aim to contribute to the expanding literature on dog–primate interactions to understand how dog attacks, with or without direct predation, can impact nonhuman primate survival in anthropogenic areas. Here we describe three cases of dog attacks (two of them probably fatal) on rhesus macaque adults in an anthropogenic landscape in North India. To our knowledge, this is the first reported case of fatal dog attacks on adult rhesus macaques.

## Study site and subjects

The cases occurred in a Hindu temple called Jakhu Temple in the City of Shimla in Himachal Pradesh in North India (31.1008° N, 77.1845° E). This site (approximately 0.02 km^2^) is frequented by around five to six groups of rhesus macaques and by hundreds of tourists every day (Kaburu et al. 2018). It consists of paved temple premises, a central garden area, a few small restaurants, a cable car tower, and a guesthouse attached to the temple grounds. Forested slopes descend from all sides of the main temple, with stairs on one slope leading down to more densely human-populated areas (for more details, see Kaburu et al. 2019). The site is also home to four to five semidomesticated or owned but free-roaming dogs. Three of the macaque groups were first monitored between 2016 and 2018 as part of a project exploring human–macaque interactions and macaque social behavior (Kaburu et al. 2018). Following that, one of those groups (Shaggy’s Group, or SG), containing 48 adult individuals (38 females and 10 males), was studied for a project on intergroup conflict, for which detailed behavioral and feeding data was collected. There were four other non-study groups whose ranges overlapped with SG, allowing us to encounter and monitor them regularly too. They were RG (females = 10, males = 5), GG (females = 7, males = 3), WG (females = 16, males = 7), and PG (females = 11, males = 8).

## Behavioral data collection:

The study group (SG) was followed from 9 a.m. to 4:30 p.m. 4–5 days a week, with detailed individual and group-level data on social behavior, feeding behavior, and human–macaque interactions being recorded. Data was collected from June 2022 to April 2023, yielding around 1157 focal observation hours. We also recorded the presence of dogs during data collection. The three cases of dog attacks on rhesus macaques reported here were opportunistically recorded using video recording and narration.

## Results

### Overall nature of dog–rhesus macaque interactions

**Fig. 1:**
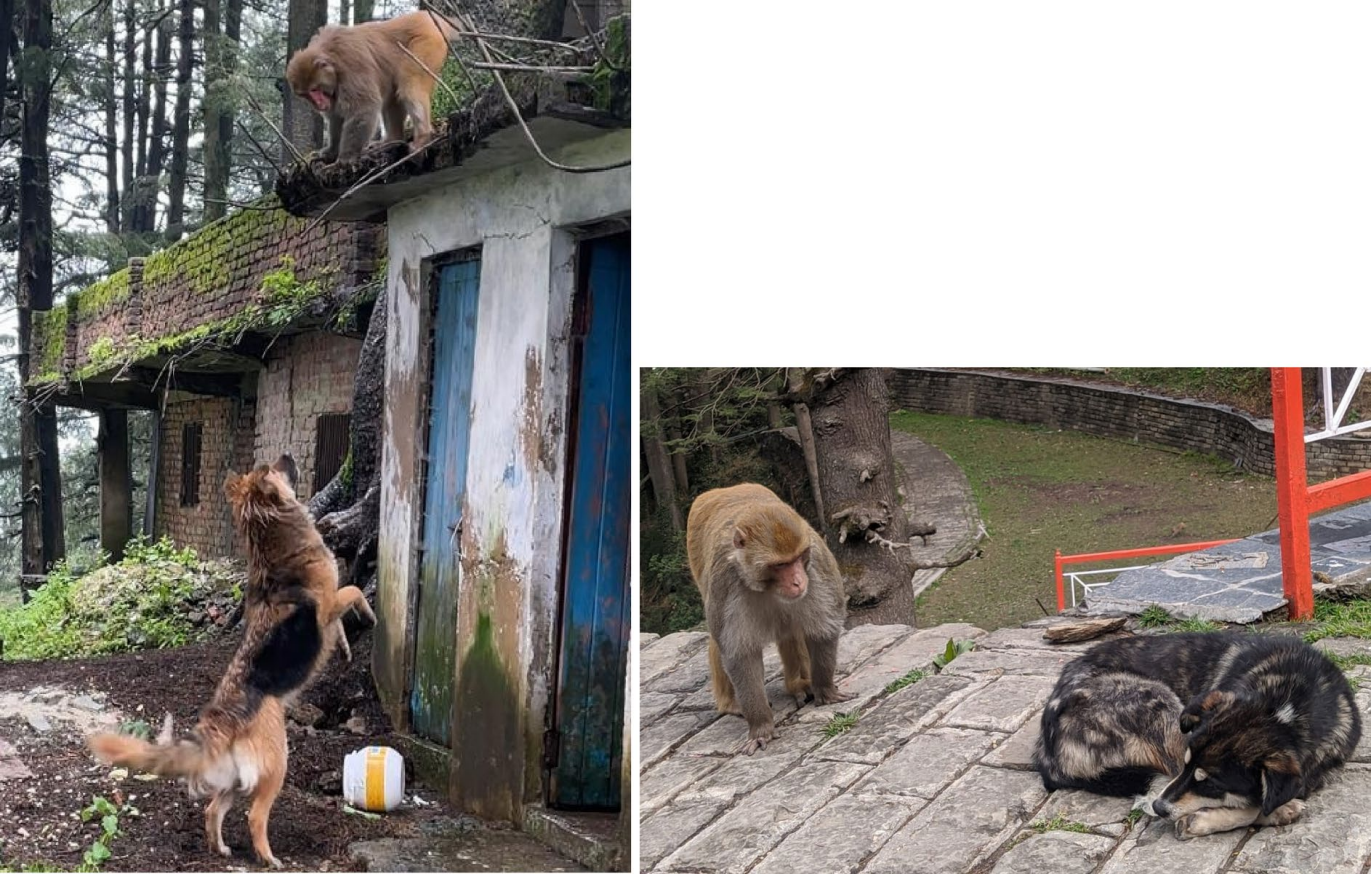
**a** (left): An interaction between a free-ranging dog and an adult male macaque. When larger dogs were around, monkeys usually sought higher ground; **b** (right) An adult male in close proximity to a smaller dog that frequents the site (photos by Subham Mohanty)

Dogs were present at the study site during observations in the period 2016–2018, but dog–macaque interactions seem to have become more aggressive since then. Four to five large dogs and one small dog were commonly observed at the site in 2022 and 2023, all except one of which belonged to a local family; the other was free-ranging. When one or more of the larger dogs were present, the macaques often ran away, emitting alarm calls and trying to climb to higher places, such as trees or temple walls (Fig. 1a). By contrast, the macaques usually ignored the smaller dog (Fig. 1b), or sometimes even chased it. Sometimes macaques threatened the dogs from a distance, even attempting to slap or scratch them, depending on whether other macaques were nearby.

**Fig. 2:**
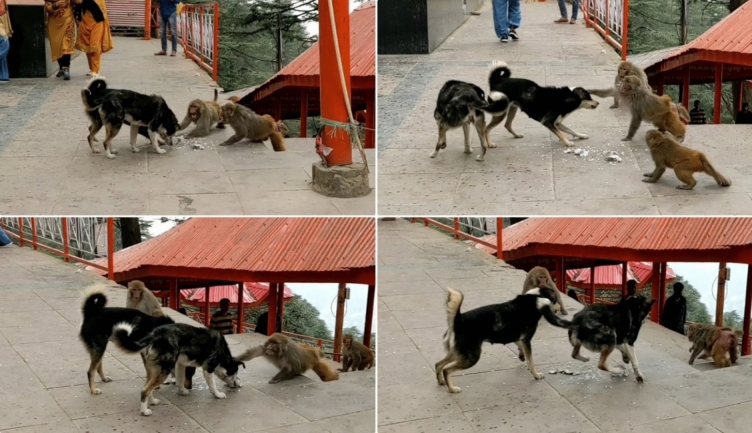
An aggressive interaction between dogs and macaques over anthropogenic food. Full video of this event is provided in Supplementary Video SV1

At this site, the dogs were mainly used by local watchmen to chase macaques away from tourists or to break up macaque fights. Every other day, whenever macaques aggregated to eat anthropogenic food, such as sugar pellets or pulses, dogs and macaques came into conflict, with dogs chasing macaques and the latter attempting to lunge at or scratch the dogs (Fig. 2). After chasing the macaques away, the dogs would often feed on the same food that was earlier being consumed by macaques. Interestingly, direct predation or consumption of the macaques by dogs was never observed, but dogs were mainly seen to chase and attack (biting and lunging) the macaques without feeding on them. Thus, it was unclear whether the interaction between dogs and monkeys at this site was mainly driven by food competition, dogs’ predatory behavior, or whether they killed the macaques opportunistically as an artifact of human intervention. The presence of dogs sometimes resulted in macaque groups vacating specific areas and staying in higher locations for up to several hours (*Supplementary video SV2*). Below, we present detailed descriptions of three dog attacks on adult rhesus macaques.

### Case 1: 31 August 2022

In May 2022, an adult female from SG (Daisy) was observed with a minor head wound, which got bigger and progressively worse over time, eventually rendering her unable to walk normally (Supplementary Fig. S1a). A few months later, on 31 August 2022 at around 4:15 p.m., we observed a fatal dog attack on this female. It took place in the parking lot of the study site, where two other non-study groups (PG and WG) were present at the time. During the attack, three dogs were chasing the monkeys, who climbed up trees on the nearby slopes. While attempting to escape from the dogs, Daisy fell from a slope onto a car from a height of 8–10 m and then tried to flee again to the slope. However, her slow speed made it easy for one of the dogs to catch her, bite, and drag her down the slope. At this time, other individuals from SG (around 15 females and 5 males), WG, and PG (unknown number) were watching the event from higher up in trees or roofs of a nearby temple and emitting alarm calls, whereas Daisy remained silent. Four mid-high ranking adult males and one mid-ranking adult female from SG, and one adult male and two adult females from PG approached the fracas, emitting loud alarm calls and looking down the slope. Given low visibility as a result of thick vegetation, Daisy’s behavior was not very clear, but the dogs did not seem to interact with Daisy or other macaques, and were eventually chased away by local taxi drivers. After 10–12 min, all three groups (SG, PG, and WG) started leaving, following which we were able to locate Daisy down the slope. She appeared to be barely alive, her hands slightly twitching, and flies settling on her head and limbs, exacerbated by her necrotic head injury. Upon getting closer to her, we were not able to see any fresh wounds apart from her earlier head injury. When the dog attack started at the parking lot, some other members of group SG (three males and six females) had escaped to the main temple located higher up the Jakhu hill and were observed looking in the general direction of the attack and making contact calls.

The next day, 1 September 2022, the corpse was found in the same place on the slope (Supplementary Fig. S1b). A few adult males from groups SG and PG separately went down to the road adjacent to the corpse and looked down the slope but did not interact with the corpse. We visited the location several times over the next few days, but were unable to locate her corpse, so it was unclear whether it was removed by local workers or dragged further down the slope by other animals.

### Case 2: 28 November, 2022

At around 12:05 p.m. on 28 November 2022, an intergroup conflict (IGC) started between group SG and group GG at a provisioning site, presumably over access to anthropogenic food. During the conflict, four dogs (three large and one small) attacked and bit an adult female from GG (*Supplementary Video SV3*). Initially, around five to six SG members and a similar number from GG were present. As soon as the female was bitten, both groups ran towards the scene and climbed into trees while emitting vocal threats as well as alarms calls. One of the larger dogs kept biting the female and had her immobilized by the neck for 20–30 sec. Any monkey that tried to approach was chased away by the other dogs. These scenes continued for 4–5 min until a local watchman chased the dogs away. The female had a gaping, bleeding wound across her thigh to her abdomen, as well as wounds on her left knee, shoulders, neck, right feet and anogenital area (Supplementary Fig. S2). Around six males (from GG) remained close to her and threatened anyone nearby, be it individuals from their group, SG, or any human observers. The female kept licking her injuries and eventually dragged herself down the slope. Two GG females approached and tried to inspect her wounds and groom her, but she slowly moved deeper into the forested slope and out of sight. During this time, males from group SG aggressively lunged at the dogs and chased them away. Soon after (around 15 min since the beginning of the event), group GG quietly moved down the slope, while group SG macaques remained uncharacteristically silent as they rested and groomed in trees and on temple walls.

The next day, we did not observe GG near the site, but one male and two females from group SG went down the slope to where the female was last seen and started emitting alarm calls. Later, we tried to go down the slope to find her corpse, but the dense vegetation and low visibility made it unfeasible. The victim of the dog attack was not seen again, and despite our monitoring the group for months following the event, no female with such extensive injuries was observed. We infer that the female succumbed to her injuries.

### Case 3: 8 December 2022

At around 10 a.m. on 8 December 2022, we witnessed an intergroup conflict (IGC) between group SG and group RG. The fight started at the main temple area and eventually spilled over onto the neighboring slopes. Initially, 13 adults and 3 juveniles were present from SG, with at least 13 adults and 8 juveniles from RG. Around 3–4 min after the IGC started, we heard alarm calls from both groups, following which three dogs ran to the area and chased the monkeys down the slopes. An RG male was attacked by a dog as he tried to descend from a tree and run down the slope. The dog bit the male’s left abdominal area, but the male broke free and escaped. Most of the macaques in the vicinity ran up trees and emitted alarm calls. This whole incident occurred over a period of a few minutes. We were unable to identify the attacked male as he swiftly escaped, and hence, we have no follow-up observations on this individual.

## Discussion

In two of the three cases described here, two adult female rhesus macaques were seriously injured and one (potentially both) died owing to dog attacks. Such observations can highlight the differential tradeoffs faced by individuals of different age–sex classes. For females, life in anthropogenic areas might be especially hazardous (Tarka et al. 2018) given their relatively small body size and the fact that they often carry dependent offspring, increasing their vulnerability to dog attacks. Moreover, injured or handicapped individuals (such as the first female from our study group) might also be especially likely to get attacked, owing to limited mobility. Reports exist of smaller and more vulnerable age–sex classes in other macaque species falling prey to dogs (Riley et al. 2015; Waters et al. 2023; Nautiyal et al. 2023). On the other hand, given their larger body size and well-developed canines, males often engage in predator defense strategies, such as alarm-calling or attacking the dogs (Nautiyal et al. 2023), which is a pattern that we also noticed at our study site.

Not only mortality from dog attacks, but the mere presence of dogs can substantially impact the socioecology of nonhuman primates, for example, negatively affecting social behavior and foraging activities (Gumert et al. 2013; Riley et al. 2015). Direct interactions with dogs and heightened anti-predator vigilance can cause physiological stress (Rangel-Negrín et al. 2023), which might eventually even have fitness effects. In fact, presence of dogs was associated with fewer juveniles in a population of long-tailed macaques (*Macaca fascicularis*) (Gumert et al. 2013). Despite the increasing evidence of dogs impacting nonhuman primate behavior and survival in anthropogenic areas, studies of whether they exert similar selective pressures as natural predators in more wild populations are required.

Observations such as ours can be used to inform discussions on the management of dog–human–nonhuman primate interactions in anthropogenic areas. Such areas often have clumped food sources, including provisioning areas and garbage dumps, where humans and animals frequently aggregate, often leading to intra- and interspecific aggressive interactions (Balasubramaniam et al. 2022). Given the fast-paced nature of these interactions, it is hard to say if the dogs at our site competed with monkeys to access anthropogenic food, or responded to the sounds of the macaques, or the temple guards’ commands to break up macaque fights. Dog–monkey interactions during foraging events in anthropogenic areas have been reported in vervet monkeys (Butler et al. 2004) as well long-tailed macaques (Riley et al. 2015). Analyzing spatiotemporal factors driving contact patterns in tridirectional interactions involving nonhuman primates, humans, and dogs might help us pinpoint hotspots of such encounters and take action to reduce or avoid the potentially harmful consequences, such as physical injuries and potential bidirectional zoonotic transfer.

We observed differences in the behavior of the group members to the deaths of two adult females killed by dogs. In the first case, Daisy had been sick for some time before the fatal attack, and her corpse received little interest from other members of her group and there were no aggressive displays by her conspecifics. Contrastingly, in the second case, the female’s traumatic and probably fatal injury elicited aggressive displays from her companions during the event. Moreover, in the former case, Daisy’s conspecifics were not observed going down to the slope where her corpse was last seen, but individuals from both SG and GG were seen going down and vocalizing on the slope where the second female was last seen. Differences in nonhuman primates’ reactions to “peaceful” versus traumatic deaths have been reported elsewhere in rhesus macaques (Buhl et al. 2012) and other species [e.g., Japanese macaques (*Macaca fuscata*) (Sugiyama et al. 2009), chimpanzees (*Pan troglodytes*) (Cronin et al. 2011), and others (Anderson 2011)] and may reflect the contrast between traumatic deaths (usually infanticide or predation) where intervention by conspecifics might be physically possible versus prolonged sickness where nothing can be done (Anderson 2011). Moreover, death of group members can affect intragroup affiliation (Buhl et al. 2012) and aggression (Kaburu et al. 2013), perhaps especially if the deceased individual occupied a socially central or dominant position in the group (Kaburu et al. 2013). The deceased female in our study group was low-ranking and socially peripheral, and we did not observe any obvious group-level changes. However, future analyses will quantitatively explore intragroup behavioral changes following her death, including any changes in the social dynamics of her affiliative partners.

Exploring diverse aspects of dog–primate interactions in anthropogenic environments is crucial for a comprehensive understanding of this phenomenon. This encompasses investigating direct predation events, assessing the impact of dogs’ mere presence on primate behavior, and recognizing the potential role of humans in shaping these interactions. Given the continual overlap of human and nonhuman animal populations worldwide, the frequency of these interactions is likely to rise. Therefore, understanding the underlying causes and consequences of these interactions becomes paramount for the survival and well-being of all organisms involved.

### Supplementary Information

Below is the link to the electronic Supplementary material.

Supplementary file1 (MP4 64534 KB)

Supplementary file2 (MP4 79505 KB)

Supplementary file3 (MP4 281314 KB)

Supplementary file4 (DOCX 857 KB)
