# Supplementary material for: Lethal dog attacks on adult rhesus macaques (Macaca mulatta) in an anthropogenic landscape
Source: Primates. 2024 Mar 6;65(3):151–7. doi: 10.1007/s10329-024-01122-y (PMC11018557; doi:10.1007/s10329-024-01122-y)
Supplement: Supplementary file 4 — Supplementary file4 (DOCX 857 KB) [file 10329_2024_1122_MOESM4_ESM.docx]

**Supplementary materials: Lethal dog attacks on adult rhesus macaques (Macaca mulatta) in an anthropogenic landscape**

Bidisha Chakraborty^1^, Krishna Pithva^1^, Subham Mohanty^1^, Brenda McCowan^1,2
1^Department of Population Health & Reproduction, School of Veterinary Medicine, University of California, Davis, CA, USA
^2^California National Primate Research Center, University of California, Davis, CA, USA

Correspondence: Bidisha Chakraborty (ORCID ID: 0000-0003-2458-0647)
Department of Population Health & Reproduction, School of Veterinary Medicine, University of California, Davis, CA, USA, email: [bchakraborty@ucdavis.edu](mailto:bchakraborty@ucdavis.edu), [bidishac02@gmail.com](mailto:bidishac02@gmail.com)

**Supplementary figures:**


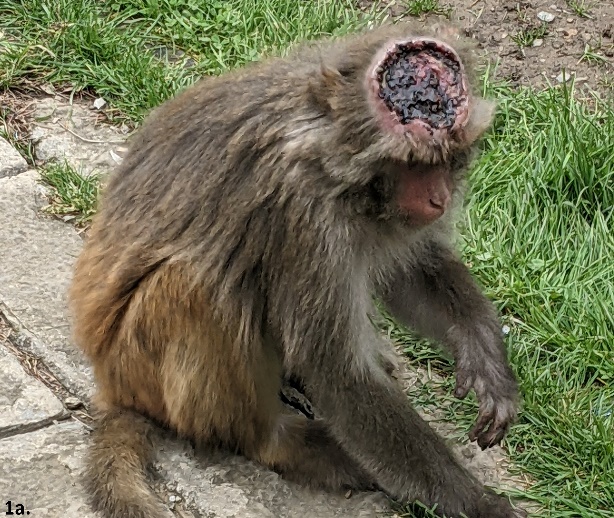

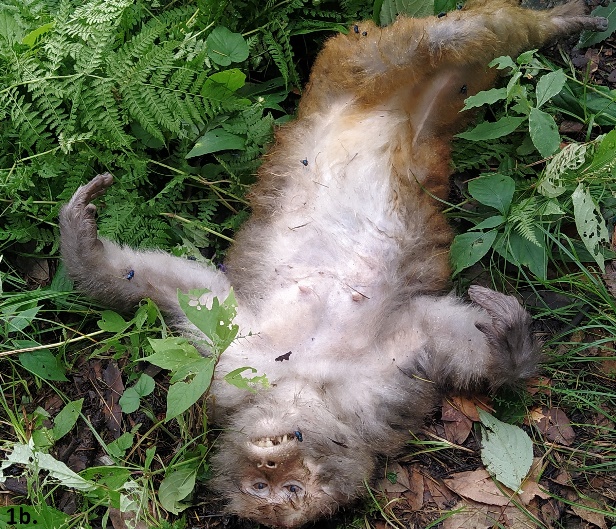
**Figure S1a:** Daisy’s injury. **Figure S1b:** The deceased female’s corpse.


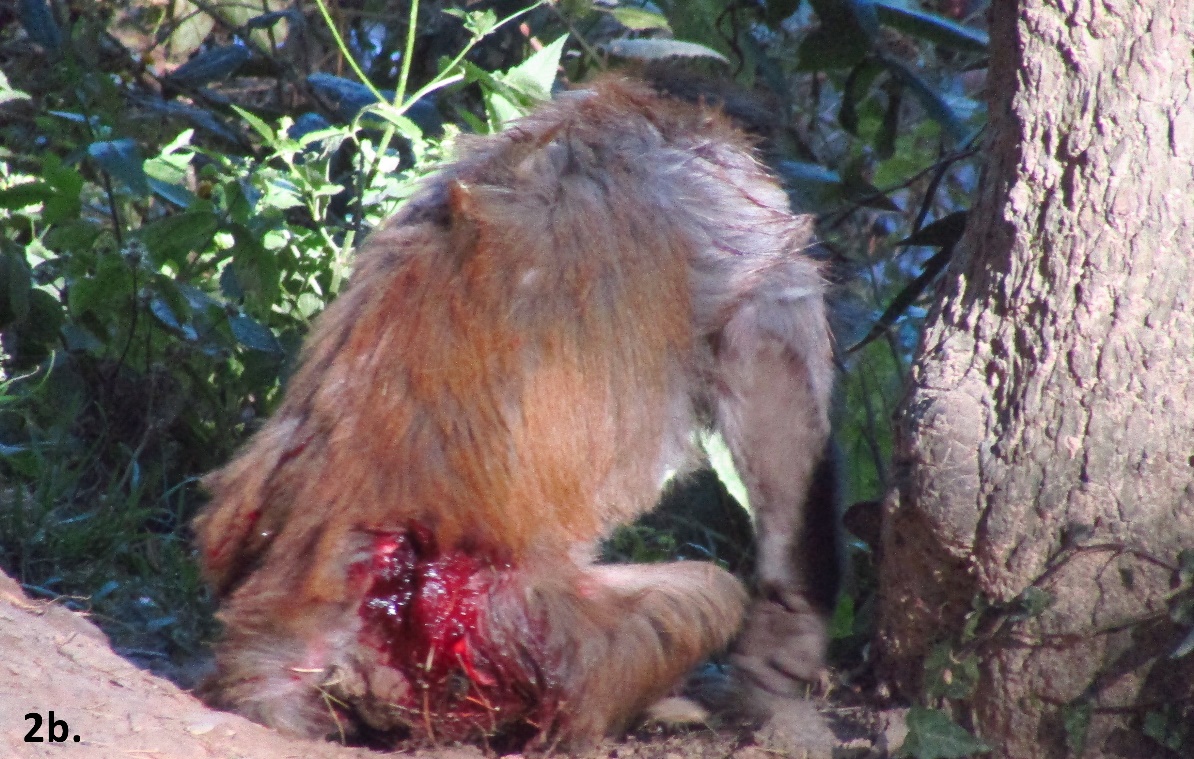

**Figure S2.** Injuries from the dog attack
